# Prognosis of AKI in malignant diseases with and without sepsis

**DOI:** 10.1186/1471-2253-13-36

**Published:** 2013-10-29

**Authors:** Malte Heeg, Alexander Mertens, David Ellenberger, Gerhard A Müller, Daniel Patschan

**Affiliations:** 1 Department of Nephrology and Rheumatology, University Hospital of Göttingen, Robert-Koch-Straße 40, 37075 Göttingen, Germany; 2 Department of Medical Statistics, University Hospital of Göttingen, Robert-Koch-Straße 40, 37075 Göttingen, Germany

**Keywords:** AKI, Sepsis, Malignancies, Mortality

## Abstract

**Background:**

AKI significantly worsens prognosis of hospitalized patients. This is particularly the case in patients with sepsis. The risk for aquiring sepsis is significantly increased in malignant diseases. Aim of the present retrospective study was to analyze outcomes of tumor patients with sepsis and AKI.

**Methods:**

One-thousand and seventeen patients, treated at the ICU of the Department of Nephrology and Rheumatology of the University Hospital Göttingen from 2009 to 2011 were retrospectively analyzed for mortality, sepsis, AKI, need for renal replacement therapy (dialysis) and malignancies.

**Results:**

AKI occurred significantly more frequent in septic than in non-septic patients and in tumor as oposed to non-tumor patients. Mortaliy rates were higher in the respective latter groups. Mortality increased even further if patients suffered from a malignant disease with sepsis and AKI. Mortality rates peaked if dialysis treatment became mandatory. In non-solid tumors 100% of the patients died if they suffered drom sepsis and AKI. This was not the case in solid malignancies (mortality rate 56%).

**Conclusions:**

We conclude that prognosis of tumor patients with AKI and sepsis is very poor. Mortality increases to almost 70% if diaylsis therapy is initiated. Non-solid tumors are associated with a 100% mortality if sepsis and AKI conincide.

## Background

Acute kidney injury is one of the major problems in today’s clinical medicine. Approximately 1-5% of all hospitalized patients develop AKI during the course of the treatment [1,2]. Prognosis has not substantially been improved during the last 20–30 years since mortality rates still vary between 30-50% [3]. The poor prognosis is not exclusively induced by AKI per se but does also result from the underlying causes / diseases leading to the decline in kidney function [4]. This is particularly the case with conditions that compromize oxygen and nutrient supply of the whole organism. Among those are heart failure and sepsis. Especially the latter has been identified as one of the most potent risk factors for AKI during intensive care treatment [5,6]. According to newer data, about 50% of all sepsis patients treated at the ICU suffer from AKI of various severity. The average mortality in sepsis-associated AKI is 50%, even if dialysis treatment has been initiated [7]. The risk for sepsis is being increased by circumstances that affect the immunological response of the host. This typically occurs in malignant diseases. The risk for infectious complications in tumor patients is dramatically increased by bone marrow-toxic chemotherapeutics. Cytotoxic treatment on the other hand can also induce AKI, depending on the type of drugs used for therapy. Maccariello and colleagues analyzed the outcome of ICU patients requiring renal replacement therapy in a prospective manner [8]. The study failed to show an association between mortality and cancer. Nevertheless, mortality was higher if patients suffered from sepsis. The authors did not separately analyze sepsis patients with a malignant disease.

Aim of the this retrospective observational single center study was to analyze outcomes of ICU patients with AKI. Thereby, our particular interest focused on mortality of AKI in malignancies with versus without sepsis.

## Methods

### Patients and setting

The present investigation was a retrospective single-centre analysis. All patients treated at the medical intensive care unit of the department of nephrology and rheumatology (University Hospital of Göttingen, Germany) between 2009 and 2011 were included into the study. It was formally approved by the local ethics committee. Acute kidney injury was defined using the AKIN criteria [9]. Patients with pre-existing ESRD (end stage renal disease) were also included into the study. In these patients, any further acute aggravation of renal dysfunction was defined as AKI if the AKIN criteria were applicable and / or if, for other reasons, dialysis treatment was initiated. Indications for dialysis were the presence of one or more of the following criteria: refractory hyperkalemia, increases of serum creatinine >3 mg/dl and / or of blood urea nitrogen >100 mg/dl at any given time point, and signs / symptoms of fluid overload due to dimished urine output, respectively. As in earlier studies [10], sepsis was defined as systemic inflammatory response syndrome (SIRS) of infectious origin [11]. Thus, beside fulfilling the criteria of SIRS [12], all patients showed at least once positive blood cultures for either Gram-positive or Gram-negative bacteria and/or clinical symptoms of an infectous disease. The term malignancy was used in any case of tumor manifestation at the time of treatment at the ICU, regardless of the respective stage of the disease. Thereby, we differentiated between solid and non-solid (hemato-oncological) malignancies. For further clinical characterization a number of different parameters, such as c-reactive protein and the SAPS (Simplified Acute Physiology Score) II scores were documented on a daily basis. All data analyzed in this study were extracted from a database, belonging to the department of Nephrology & Rheumatology of the University Hospital of Göttingen.

### Statistical analysis

All results are expressed as percentages. Differences between 3 or more groups were analyzed by ANOVA. Differences between two groups were analyzed by chi-square test. Significance was considered at p<0.05.

## Results

### Patients

A total of 1.017 patients were included into the study. Six-hundred and eight were male, 409 were female, the mean age of all patients was 65 ±16 years with 65 ±14 years in men and 66 ±18 years in women. All patients were treated at the intensive care unit of the department of nephrology and rheumatology of the university hospital Göttingen (Germany) between 2009 and 2011. Sepsis was diagnosed in 330 patients (32% - 208 male [63%], 122 female [37%]), 687 patients (68%) did not fulfill the respective criteria. Two-hundred and twelve patients (21% - 138 male [65%], 74 female [35%]) suffered from a malignant disease at the time of admission to the ICU (non-solid tumor: 88, solid tumor: 124). Thirty-three patients with a non-solid tumor underwent bone marrow-/stem cell transplantation in their history. Four-hundred and thirty-five patients (43% - 278 male [64%], 157 female [36%]) either presented with AKI at the time of ICU admission or developed AKI during the treatment course at the ICU. Liver cirrhosis was diagnosed in 83 patients (8% - 57 male [69%], 25 female [31%]). The most important general outcome parameters of all included patients are summarized in Tables 1 and 2.

**Table 1:** Outcome characteristics

**Variables**	**Survivors**	**Non-survivors**	**p-value**
Age	64.3 ±16.6 years	69.5 ±13.5 years	<0.001
Male gender	440 (58%)	168 (64%)	0.066
SAPS II	30.4 ±10.7	41.8 ±10.9	<0.001
AKI	287 (37%)	148 (56%)	<0.001
Sepsis	175 (23%)	156 (60%)	<0.001
AKI+Sepsis	105 (16%)	117 (45%)	<0.001
Cancer	135 (17%)	76 (29%)	<0.001
Liver cirrhosis	52 (7%)	30 (12%)	0.017
Dialysis	224 (30%)	120 (46%)	<0.001
ICU stay	7.4 ±8.1 days	7.4 ±8.4 days	0.9
Controlled ventilation	244 (32%)	185 (71%)	<0.001

Table 1 compares frequencies of different variables / mortality risk factors between survivors and non-survivors. The differences between the two groups were significant in all analyzed categories, with the exception of male gender and length of ICU stay. Table 1 shows frequencies of the three AKIN stages in AKI patients if used for diagnosis, followed by the respective mean serum creatinine concentrations at the time of admission to the ICU (results as mean ±SEM).

**Table 2:** Serum creatinine concentrations in AKI patients before onset of AKI

**AKIN stage**	**n**	**[Creatinine]**_**S **_**before onset of AKI (mg/dl)**
1	27	1.3 ±0.1
2	14	2.4 ±0.3
3	95	3.7 ±0.2

### AKI in sepsis

**Figure 1:**
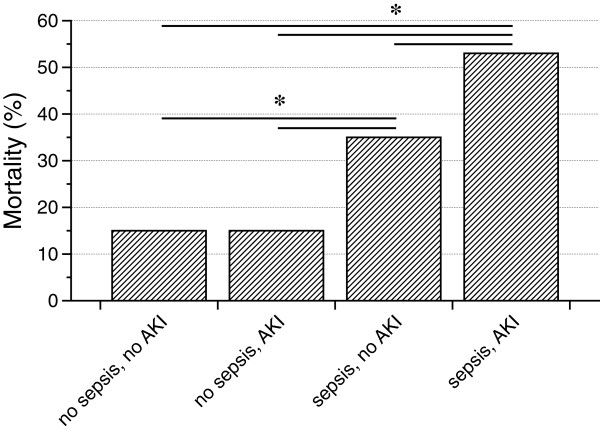
**Mortality in septic versus non-septic patients with and without AKI.** The prognosis did not differ between non-septic patients with or without AKI, mortality was 15% in both groups (no AKI 72/474, AKI 31/213). Septic patients had higher mortality rates as oposed to those without sepsis, independently from the presence of AKI (sepsis without AKI – 36% (39/108), sepsis with AKI – 53% (117/222)) (✻: p<0.001).

As pointed out earlier sepsis was diagnosed in 330 patients (32% - 208 male [63%], 122 female [37%]), 687 patients (68%) did not fulfill the respective criteria. In the no sepsis group 474 patients (69%) did not develop AKI while 213 (31%) presented with AKI of various severity. One-hundred and eight patients (32%) in the sepsis group did not suffer from AKI during the course of the disease, but AKI developed in 222 individuals (68%). Thus, AKI occured significantly more frequent in sepsis than in non-septic patients (p<0.001). The mortality rates within the respective groups were: no sepsis without AKI - 15% (72/474), no sepsis with AKI – 15% (31/213), sepsis without AKI – 36% (39/108), and sepsis with AKI – 53% (117/222). Mortality rates were significantly higher in patients with sepsis (± AKI) as compared to those without sepsis (± AKI). Additionally, patients with sepsis and AKI died more frequently than those wihtout AKI (Figure 1).

### AKI in malignancies

**Figure 2:**
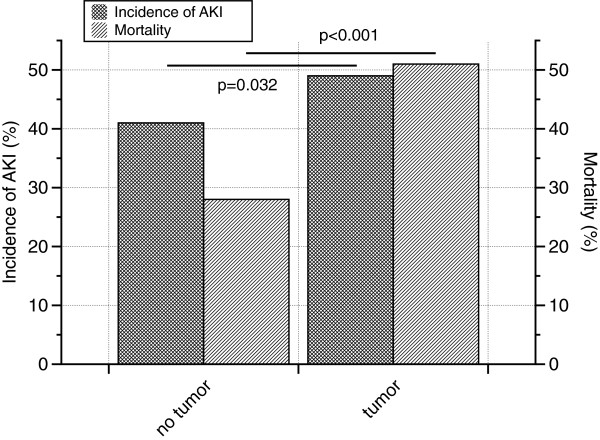
**Incidence of AKI and mortality in tumor versus non-tumor patients.** Incidences of AKI were: patients without malignancy 329/805 (41%), patients with a malignant disease (either solid or non-solid tumor) 104/212 (49%). The difference was statistically significant (p=0.032). Mortalty rates were: patients without tumor but with AKI 93/329 (28%), patients with malignancy and AKI 53/104 (51%). The calculated p-value was below 0.001.

Two-hundred and twelve patients (21% - 138 male [65%], 74 female [35%]) suffered from a malignant disease at the time of admission to the ICU. A solid tumor was diagnosed in 123 patients (58%) while a non-solid tumor was apparent in 87 patients (42%). Incidences of AKI were: patients without malignancy 329/805 (41%), patients with a malignant disease (either solid or non-solid tumor) 104/212 (49%). The difference was statistically significant (p=0.032). Mortalty rates were: patients without tumor but with AKI 93/329 (28%), patients with malignancy and AKI 53/104 (51%). The calculated p-value was below 0.001. Thus, the coincidence of a malignant disease and AKI dramatically worsened the overall prognosis (Figure 2).

### AKI in malignancies with sepsis

**Figure 3:**
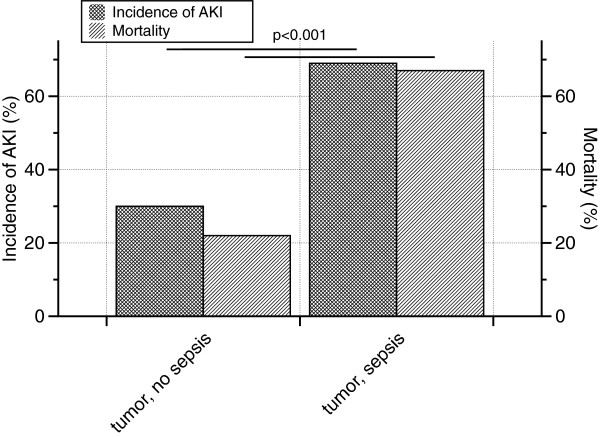
**Incidence of AKI and mortality in tumor patients with versus without sepsis.** One-hundred and fifteen patients with a malignant disease did not suffer from sepsis (54% - 115/212). The incidence of AKI was 30% (35/115) and mortality was 22% (8/35). Ninety-seven patients with a malignancy were diagnosed with sepsis (46% - 97/212). AKI occurred in 69% (67/97), mortality was 67% (45/67).

One-hundred and fifteen patients with a malignant disease did not suffer from sepsis (54% - 115/212). In this group, the incidence of AKI was 30% (35/115) and mortality was 22% (8/35). Ninety-seven patients with a malignancy were diagnosed with sepsis (46% - 97/212). In this particular group, AKI occurred in 69% (67/97), mortality was 67% (45/67). In both categories (incidence of AKI, mortality) differences between the two groups (tumor with vs. without sepsis) were significant (p-values lower than 0.001 - Figure 3). In a subgroup analysis, mortality rates of patients with sepsis and AKI were compared, depending on the presence of a solid versus non-solid tumor disease. As a matter of fact, patients with a non-solid tumor died in 100% if sepsis and AKI coincided, as compared to those with a solid tumor disease. In the latter group, mortality was 56% (p<0.001).

### Mortality and dialysis

A total number of 341 patients required dialysis treatment. Subgroup analysis revealed the following frequencies of renal replacement therapy in patients with versus without malignant diseases: patients without tumor and without sepsis (28%) 94/341, mortality was 15% (14/94), patients without tumor but with sepsis (28%) 95/341, mortality was 46% (44/95), patients with tumor and without sepsis 6% (20/341), mortality was 20% (4/20), patients with tumor plus sepsis 11% (36/341), mortality was 66% (24/36). The following differences in mortality rates were statistically significant between the groups: no tumor, no sepsis versus no tumor, sepsis p<0.001, tumor, no sepsis versus tumor, sepsis p<0.001, no tumor, sepsis versus tumor, sepsis p=0.037.

## Discussion

Aim of this study was to retrospectively analyze epidemiology and outcome of ICU patients with AKI. Our particular interest focused on AKI mortality rates in malignancies and sepsis.

AKI significantly worsens the prognosis of hospitalized patients [2,4]. This is particularly the case in the ICU setting in which mortality can increase to 60% [4]. Meanwhile, sepsis / septic shock have been emerged as the most frequent causes of AKI at the intensive care unit [6]. The pathogenesis of sepsis-associated AKI is complex and includes severe hemodynamic alterations with subsequent renal hypoperfusion on one hand, but also systemic activation of the innate and aquired immune response leading to inflammation of the kidney on the other hand [6,13]. The processes involved shall not be reviewed more in detail at the moment. However, regarding the widespread host-initiated defense mechanisms it becomes understandable that mortality rates gradually increase with progressive severity of the septic syndrome [14]. The prognosis of sepsis-associated AKI also depends on the severity of renal dysfunction *per se*: Bagshaw and colleagues showed higher AKI mortality rates in septic shock with progressive deterioration of renal function [15]. Interestingely, the authors did not find higher incidences of AKI if the diagnosis of metastatic solid organ cancer was made [15]. Patients with non-solid hematologic malignacies on the other hand developed AKI more frequently. Finally, Plataki and colleagues identified numerous AKI risk factors in septic shock, including delayed initiation antibiotic therapy, intra-abdominal sepsis, and blood product transfusion [16]. A detailed metaanalysis about AKI risk factors in sepsis was published by Cartin-Ceba et al. in 2012 [17].

As oposed to sepsis, there are generally less data available about epidemiology and outcome of AKI in tumor patients. A recently published cross-sectional analysis of prospectively collected data revealed an AKI incidence of 12% in the latter group of patients [18]. In this study AKI risk was significantly correlated with pre-existing diabetes, hyponatremia, intravenous contrast media administration, chemotherapy, and antibiotics. Sepsis in contrast was not identified as risk factor. Another study evaluated predictors of hospital mortality in critically ill cancer patients according to the severity of renal dysfunction. AKI, as defined by the RIFLE criteria [19], occurred in 54.2% and mortality increased with progression from ‘R’ to ‘F’ [20]. The results from our study are more or less in line with data from the literature: incidences of AKI were higher in sepsis than in non-septic patients, survival in sepis-associated AKI was lower than in AKI without sepsis or in sepsis without AKI. Frequencies / survival of AKI were higher / lower if a malignant disease was present. Somehow surprising was the observation of comparable mortality rates in non-tumor, non-septic patients with versus without AKI (15%). One might argue that due to the absence of sepsis, the overall morbidity of these patients was lower than in septic individuals.

The first new aspect in our study is related to the conincidence of tumor and sepsis and to survival rates of these patients if dialysis therapy became mandatory. In the latter group, mortality increased to almost 70% (66%) which once had been reported as the average mortality of AKI patients in the 1970s [21]. A second new aspect is related to mortality in tumor patients with sepsis and AKI, regarding the respective nature of the malignant disease. If a non-solid tumor was diagnosed, mortality was 100% as compared to patients with non-solid malignant disease. In those patients mortality was significantly lower (56%). To our knowledge, this observation has never been reported before and we can only speculate whether non-solid tumors affect the outcome by mechanism related to the disease *per se* or if the poor outcome results from more aggressive therapeutic interventions. In summary, we conclude that prognosis of tumor patients with AKI and sepsis is very poor. We are well aware of the fact that due to the retrospective and single center-based character of our study, conclusions must be drawn with caution. Thus, further prospective analyses are urgently needed.

## Conclusions

We conclude that prognosis of tumor patients with AKI and sepsis is very poor, with a mortality of approximately 70% if diaylsis therapy becomes mandatory. Mortality increases even further if patients suffer from a non-solid malignant disease, in this particular group almost 100% of the patients die.

### Key messages

•Sepsis and AKI significantly worsen prognosis of tumor patients.

•Dialysis treatment in this group is associated with an average mortality of 70%.

•Non-solid tumors are associated with mortality rates of 100% if patients suffer from sepsis and AKI.

## Abbreviations

AKI: Acute kidney injury; ESRD: End-stage renal disease; ICU: Intensice care unit; RIFLE: Risk injury failure loss end-stage renal disease; RRT: Renal replacement therapy; SOFA: Sequential organ failure assessment.

## Competing interests

The authors declare that they have no competing interests.

## Authors’ contributions

MH designed the study and analyzed data. AM collected all data. DE helped in statistical analysis. GAM corrected the manuscript. DP analyzed the data and wrote the manuscript. All authors have nothing to disclose. All authors read and approved the final manuscript.

## Pre-publication history

The pre-publication history for this paper can be accessed here:

http://www.biomedcentral.com/1471-2253/13/36/prepub
